# Shocking Data on Parcel Shipments of Protein Solutions

**DOI:** 10.1016/j.xphs.2019.10.064

**Published:** 2020-01

**Authors:** Christine Siska, Paul Harber, Bruce A. Kerwin

**Affiliations:** 1 Just Biotherapeutics, Inc., Seattle, Washington 98110; 2 Parenteral Supply Chain, LLC, Indianapolis, Indiana 46208

**Keywords:** antibody, biotechnology, developability, formulation, high concentration, protein formulation, protein structure, protein folding, protein aggregation, physical stability

## Abstract

An early-phase development shipping study was designed to interrogate the stability of liquid formulations under normal shipping conditions. Parcel shipments were made between Seattle, WA, and Indianapolis, IN, during 2018-2019. Each parcel contained a data recorder that tracked the shipment by GPS and measured shock and temperature. During the transport process, the parcels received up to 40 shock events with strengths ranging from 8 to 36G. After shipment, the formulations without polysorbate showed considerable increases in submicron and visible particles while little to no change occurred when polysorbate was present. Samples dropped repeatedly from a height of 18 inches to produce a shock of ∼25G caused visible particle formation with little increase in the subvisible particles, suggesting that other factors, such as vibration, in addition to the shock, were necessary to produce particle formation. These results provide a basis for further studies in the relationships between physical stability of mAbs and the challenges introduced by the shipment network, specifically shock and vibration. The findings indicate that the shock events as measured are repeatable and attributable to the layout of the sorting facility.

## Introduction

Although extensive care is taken to manufacture and package biologics in a highly controlled process, shipping these products to distribution centers, pharmacies, and patients is carried out with minimal control once it leaves the manufacturing facility. Shipping containers are designed and validated to maintain constant temperatures in defined ranges and prevent high temperature excursions known to degrade biologics but are not designed to dampen or prevent shock exposures which occur at multiple points during distribution, potentially damaging sensitive biologic products. Shock events may cause aggregation and both subvisible and visible particle formation leading to immunological events upon injection of the drug product. The immunological events may encompass multiple problems from simple injection site reactions to more serious problems such as immunogenic reactions leading to antidrug antibodies.^1^, ^2^, ^3^

The shipping process can be broken up into a series of steps, each causing unique stresses on the drug product.^4^ The bulk drug substance is not considered here because it is generally shipped frozen and does not experience the stress associated with shipping liquid products. Other than human serum albumin which can be stored and shipped at room temperature, all other biologics are stored and shipped either frozen or at 2°C-8°C. The stages can include ground transportation, air transportation, distribution center storage, and delivery to the hospital, pharmacy, doctor’s office, or patient. Within each stage, the primary stresses include vibration and shock but may also include temperature excursions, light exposure, and air pressure changes.^5^ An additional factor overlaying these stresses is the amount of time the stress is applied and the strength and number of shock events each product will experience. The combination of these factors leads to the physical degradation of the drug product.

All shipments are exposed to vibration energies and the base level of the profiles for the various modes has been well documented^6^ with the vibration frequency dependent on the type of transport and the suspension system used such as spring suspension truck, air ride suspension truck, rail line, or aircraft transport. Palletized product directly transported from point A to point B in a dedicated trailer will be exposed to vibration and to a lesser extent, shock energies. Indirect transport of parcel shipments through a node and hub network such as FedEx is exposed to vibration, and significant shock events at the sort hubs. Vibration frequencies are in the range of 3-300 Hz with higher energy levels observed at the top level of a pallet compared with the lower level.^7^ Although the increased vibrational frequency is known to cause greater damage to hard goods such as lawn mowers and water heaters, the effect of the vibrational frequency on protein solutions is not clear. Prior work by Fleischman et al.^8^ showed that use of a table top vibration table caused aggregation to a level similar to that seen for real-time shipping studies between Maryland and California. Subvisible particles were the main degradation route with no mention of visible particle formation. It is possible this was due to the use of accelerated vibration profiles used for the laboratory simulation. Based on American Society for Testing and Materials standards,^6^ typical vibration levels are accelerated by a factor of 5× compared with that which may be recorded in the field. The vibration profiles are accelerated to reduce the time on the lab simulator. Although this type of acceleration has proven useful for hard goods, it is not clear how this affects protein solutions or if this would translate to real-world shipping experience. A recent report by Wallin^5^ states that “Amgen does not accelerate its random vibration profiles and executes test durations 1:1 with expected transportation time. Amgen studies have shown our laboratory test data do not match actual shipment data when accelerated testing is performed.”

Shock-induced cavitation is the suspected mechanism that leads to physical degradation. Multiple studies have shown that uncontrolled shock events during the shipping process can cause particle formation in the drug product.^9^^,^^10^ Shock events can occur during the shipping process because of accidental drops, loading of vials on trucks, airplane landings, and other unforeseen events. Cavities form in solution because of a shock wave; their rapid expansion followed by violent collapse creates short-lived hot spots that can reach thousands of degrees Kelvin and hundreds of atmospheres.^11^^,^^12^ Randolph et al.^9^ showed that cavitation bubbles can form within 30 μs of shock event resulting in hydroxyl radicals leading to gelatinous particles forming on the vial walls and a subsequent increase in subvisible particle formation in solution. This occurred with drop heights as short as 10 inches but was dependent on the protein concentration. Torisu et al.^10^ reached similar conclusions after multiple cycles of dropping and shaking and also showed the degree of particle formation was also dependent on the volume in the vial. In other studies of cavitation formed by flow through a microorifice, Duerkop et al.^13^ concluded that the increased surface area alone was responsible for observed aggregation of human serum albumin and not due to hydroxyl radical formation. This suggests that multiple factors including small shock events during shipping or handling may result in degradation of the drug product and the final observations of aggregation and particle formation may be product dependent.

Many companies rely on simulated shipping studies to verify the physical stability of the formulation during development. At the time of Biological License Application submission, product data from properly conducted simulated studies are submitted and compared with data from the registration batches of drug product. Simulated shipping studies take both the vibration and the shock components into account within a controlled environment to understand what may affect the stability of the product.^5^^,^^14^ In addition to vibration and shock, other variables are controlled including time, temperature, and air pressure. Air pressure exposures must be considered for product filled into syringes and cartridges. These containers will likely entrap an air bubble, and movement of the unrestrained plunger is to be expected. For phase 3 and commercial formulation programs, it is important to carry these studies out in a well-controlled environment to fully understand the combined effects of the different variables on the drug product stability. For early development, however, when a variety of formulations are being tested, a fast, realistic, and inexpensive method is desired. Although a number of laboratory methods have been used including shaking stress,^15^, ^16^, ^17^, ^18^ vortexing stress,^19^, ^20^, ^21^ and compression/dilation stress^22^ to simulate continual formation of the air-liquid interface, it has been difficult to correlate the results of these methods to actual shipping conditions.

This article is intended to examine the effect of vibration and shock events on an array of formulations. Of particular interest is determining the number and intensity of shock events within a parcel network so that an accurate replication of these events can be performed within a simulated study. Samples of the mAb, Ustekinumab, were shipped round trip at refrigerated temperature from Seattle, Washington, to Indianapolis, Indiana, with a SenseAware® PT300D tracking device that records temperature, journey coordinates, and shock events including the time, place, and force of the shock. The results demonstrated that a consistent number of shocks with similar force occurred at defined points throughout the shipping process. Comparing shipped to nonshipped samples, we were able to demonstrate that, as expected, polysorbate was necessary to protect the protein from aggregation and particle formation. Surprisingly, when simple drop testing was performed from 18 inches without the additional shipping components, neither subvisible particle nor visible particles were observed, suggesting that in our studies, both shock and vibration were necessary to fully stress the samples and observe degradation.

## Materials and Methods

### Materials

#### mAb Sample Preparation

mAb 1, ustekinumab, was provided by Just Biotherapeutics, Inc. and was formulated at 90 mg/mL with 20 mM sodium acetate, 9% (w/v) sucrose at pH 5.2. Formulations contained polysorbate 80 (NFP Corporation) at concentrations of 0%, 0.01%, 0.02%, or 0.03% (w/v), which were added from freshly prepared 2.0% (w/v) stock solutions prepared in milliQ water. All formulations were sterile filtered (0.22 μm Sterivex filter) in a laminar flow hood, and aliquots, 1.5 mL, were dispensed in triplicate into ISO2R Top Line vials (Schott), sealed with flurotec-coated butyl rubber stoppers (Daikyo), and metal crimp seals (West). The vials can hold up to 3 mL so that a large air gap is present in the vial configuration used here.

#### Packaging

Vialed samples were packaged in a sample box designed to hold 100 vials (Stephen Gould). To ensure the weight of the sample box was representative of a drug product shipment, empty spaces were filled with vials containing 1.5 mL of water. Sample boxes were placed in a NanoCool® shipping system (FedEx) that consists of a qualified cold shipping package equipped with a 48-h duration cooling engine.

#### Shipping and Monitoring

All shipment boxes contained a SenseAware PT300D monitoring, FedEx, set to monitor location and shock events above 5G. The boxes were shipped FedEx priority overnight from Seattle to Indianapolis. Each trip was picked up in downtown Seattle, Washington, by FedEx and delivered to a FedEx storefront in Indianapolis, Indiana. On route to Indianapolis, the package moved through a sorting hub in Memphis Tennessee. Once in Indianapolis, the package was delivered to a FedEx storefront at which point the shipping box was subjected to a series of 10, flat-bottom drops from 18” and returned to Seattle. The return routing to Seattle was direct and did not go through the Memphis sort hub.

Samples were maintained at 2°C-8°C using a NanoCool® packaging system and monitored with a SenseAware device. The Nanocool device was activated during sample packaging, and the desired temperature was reached within 1 h. The box remained at approximately 5°C for the duration of the 48-h shipment (data not shown). Each shipment was conducted at a separate time over a 9-month period.

#### Visible Particle Inspection

Samples were inspected for visible particles against a black background under a 60W incandescent bulb with an illuminance of 9000 lux.

#### Subvisible Particle Analysis

Subvisible particles were measured using a FlowCam 8100 benchtop microflow imaging system equipped with an 80 μm flow cell and a 10× magnification lens and controlled by the Visual Spreadsheet software. Triplicate vials were equilibrated to room temperature and gently swirled to mix thoroughly. Single readings of 100 μL from each vial were collected, and total particle concentration above 2 μm was recorded.

## Results and Discussion

**Figure 1:**
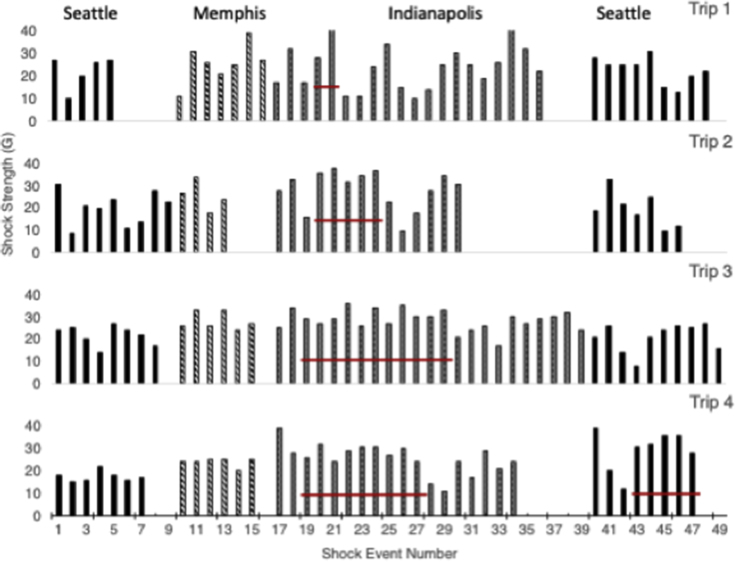
Shock strength monitoring. Summary of the shocks that were recorded for 4 test shipments. The solid bars are shocks recorded in Seattle, the dashed bars are shocks recorded in Memphis, and the checkered bars are shocks recorded in Indianapolis. The red line indicates the shocks were performed intentionally by the recipient in Indianapolis, and in the case of trip 4, the recipients in Indianapolis and in Seattle. Because the monitoring system records data through a cell network, unavailability of the network will lead to underreporting and may have occurred here.

Over the course of the shipment, the package was monitored for the strength of each shock as the box was moved or dropped. Figure 1 contains a summary of the shocks that were recorded by the SenseAware data recorder for 4 test shipments. The solid bars are shocks recorded in Seattle, the dashed bars are shocks recorded in Memphis, and the checkered bars are shocks recorded in Indianapolis. The red line indicates the controlled 18” drops performed intentionally by the recipient in Indianapolis. These 10 drops were designed into the first shipment based on the need to verify the American Society for Testing and Materials and International Safe Transit Association standards in place for testing drop heights in parcel networks. Because we designed these into the first study, we decided to keep the process constant in the other shipments as well. In retrospect, the additional flat drops were not necessary for product exposure because of the number and strength of the shocks that occurred throughout the process. The consistency of the shock data across the 4 shipments as the container traveled through the network data showed a repeatability of the drop events and strongly suggests that the shocks are not random. It also exposed an issue with the SenseAware® system concerning the capability to record multiple events that may occur over a short time period. Although 10 flat drops were performed from 18 inches in Indianapolis in shipment #1, only 4 were actually recorded by the device. When a shock event occurs, the SenseAware unit apparently locks the data buffer and immediately attempts to write the shock event to a cloud-based server over a cell network; therefore, if the events occur too frequently then subsequent events may not be recorded. We also found that when flat drops were performed in slow enough succession to record the drops if they occurred in an area with a poor mobile phone signal, then fewer of the drops were recorded as well. Together this suggests that not all shock events may have been recorded by the device and that a higher number of shocks occurred during the shipping process. A better system to use would record the shocks and store them on the device so that all events could be detected. It is also possible that fewer than the stated shock events were recorded because of our setting the threshold at 5G. Because we do not truly know the importance of slower accelerations, it may be better to set the device to record shocks at accelerations above 1G to fully understand the extent of events that occur throughout the shipping process.

Based on the data from the shipping studies, a number of observations can be made. First is that the shocks are variable across each leg of the shipment. The acceleration of the shocks ranges from a low of 10G to a high of 40G with a mean of 24.6G. Across the 4 shipments performed for this study, 4% of the shocks were between 5 and 10G, 24% were between 10 and 20G, 50% were between 20 and 30G, and 22% were between 30 and 40G. Within each stage, the shock accelerations were variable, likely representing those due to loading, transport, sorting, and unloading of the container. At the Memphis facility, the number of shocks was fairly consistent ranging from 4 to 7 events with 3 of the shipments showing 6-7 shock events each. This would be consistent with the containers going through a similar sorting path each time they went through the Memphis hub. For other trip segments, the number of shocks recorded varied with ranges of 5-9 for the first Seattle segment, 9-18 for the Indianapolis segment, and 3-10 for the second Seattle segment.

**Figure 2:**
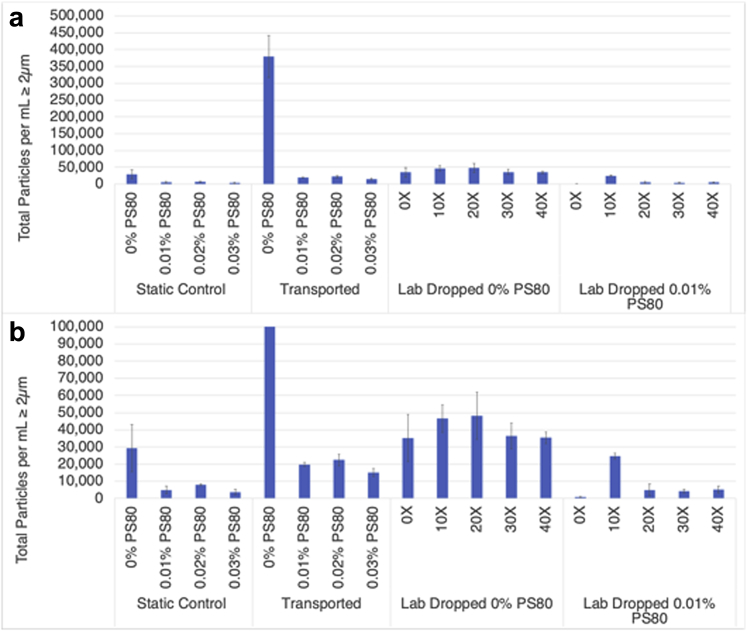
Subvisible particles. Subvisible particles were counted for each sample included in Trip 4. The total particles per mL 2 μm and more for each condition included in the shipment are shown. Panel a is the plot of all data, and panel b is the zoomed in view of the data shown in panel a.

Subvisible particles were measured for each sample included in Trip 4. Figure 2 contains the total particles per mL ≥2 μm for each condition included in the shipment. The samples that experienced an actual shipment contained significantly more subvisible particles than those samples that were held statically at 2°C-8°C. Adding polysorbate in concentrations typically found in protein formulations, 0.01%-0.03% w/v,^23^, ^24^, ^25^ reduced the subvisible particle content in all conditions tested with a slight reduction in particle content for the 0.03% w/v sample as compared to those with 0.01% w/v polysorbate 80. In addition, although an increase in particle content was observed for all samples which underwent shipping, the highest polysorbate concentration showed a slight decrease compared to that at 0.01%.

**Figure 3:**
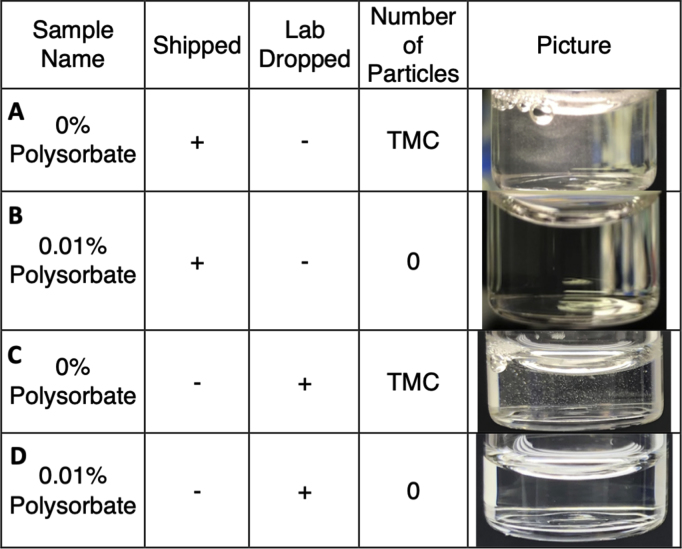
Visible particles in shipped and lab dropped vials. Individual particles are counted up to 50 particles, if more than 50 particles are present TMC (too many to count) is recorded. Plates a and b were from shipping study trip 4, whereas plates c and d were after 40 drops in the laboratory from a height of 18 inches. Plate a: formulation with no polysorbate, plate b: formulation with 0.01% w/v polysorbate 80, place c: formulation with no polysorbate, plate d: formulation with 0.01% polysorbate 80.

Similar observations were made for the visible particles in which a cloud of particles was apparent in the shipped vial without polysorbate while the shipped vial with 0.01% polysorbate was free of visible particles (see Fig. 3). This result is in agreement with many previous studies demonstrating that polysorbate effectively prevents subvisible and visible particle formation during simulated shipping.^8^^,^^10^ This effect may be due to the polysorbate binding hydrophobic regions of the antibody,^26^ the polysorbate outcompeting the antibody for access to the air/liquid interface,^17^^,^^27^^,^^28^ or a combination of both. Other studies using both real and simulated shipping have neither shown nor discussed visible particle formation making it unclear if these may have formed, albeit the studies did show large increases in subvisible particle formation. The work by Randolph et al.^9^ showed formation of a gel layer in the vials after repeated drops, but it was not clear if this could lead to visible particle formation. A similar observation was made by Torisu et al.^10^ It is entirely possible that particle formation is molecule specific and that the particular antibody used for our studies readily forms visible particles with shipping stress and that those molecules used in the previously mentioned studies do not.

A final set of experiments were conducted to investigate subvisible and visible particle formation due strictly to drop shock events without the additional vibration stress experienced during shipping. In this study, the package was repeatedly dropped from the standard 18 inch height at regular intervals and the drop acceleration values, subvisible particles, and visible particle were measured. The shock range for the drops varied from a low of 31G to a high of 41G with an average value of 36G. These values are similar to those measured during the round trip shipments (Fig. 1). Measurement of subvisible particles after the drops showed a distinct difference in number compared with shipped samples. Relative to the control (0× dropped sample), the vials dropped 10×, 20×, 30×, and 40× did not increase significantly in subvisible particle counts, whereas the shipped sample without polysorbate increased approximately 10-fold (Fig. 2). Visible particles in the dropped only sample without polysorbate were readily apparent after 10, 20, 30, and 40 drops, whereas no particles were present in the sample containing polysorbate (Fig. 3). Although particles were present in the shipped and dropped only samples, the shipped samples showed a significant increase in the number of visible particles with the shipped samples showing a cloud of particles that were too many to count and the dropped only samples showing distinct particles in solutions. The difference in the overall results of the subvisible and visible particles between the shipped and dropped only samples suggests that with this particular molecule, we can partially replicate the instability observed during shipping but are not accounting for all stresses leading to the instability. The obvious difference here is the absence of vibration stress before or after the shock events. The vibration stress alone or in combination with the shock events may be what caused the increased subvisible and visible particle formation or there may be unknown stresses that are not accounted for. Future studies in our laboratories incorporating a vibration table along with the drop/shock events will help establish the connection. Although our data are in contrast to those of Randolph et al.^9^ in which shock events were associated with large increases in subvisible particle formation, and Torisu et al.^10^ in which synergetic effect enhances protein aggregation, it may be that we are observing product-specific effects that originate from the same basic process as previously described but follows through to a different end point because of the nature of the antibody and conditions tested here.

## Conclusions

The studies described here demonstrate the high degree of variability in the shock events experienced by the drug product containers during shipping. Although the studies are relatively uncontrolled compared with using vibration tables and drop systems, the number of shocks and accelerations are relatively consistent. By our measurements, up to 40 events occurred during the shipping studies described here with a much higher number likely occurring if the acceleration threshold was set to a value less than 5G. To our knowledge, this is the first study showing the number, acceleration, and location of the events during the shipment of a biologic. As shown by prior work of other investigators,^9^ only a single shock event was necessary to cause aggregation in a liquid drug product. The study also shows that while polysorbate significantly reduced the degree of subvisible particle formation, they do not completely prevent its formation, albeit the polysorbate did prevent visible particle formation for the antibody studied here. This information, in combination with understanding the role of vibration and vibration frequency, can help establish improved simulated shipping protocols.
